# The -124C>T Mutation of the *TERT* Promoter Indicates Favorable Prognosis in Ovarian Clear Cell Carcinoma: A Single Institutional Study in China

**DOI:** 10.3390/curroncol32080422

**Published:** 2025-07-27

**Authors:** Xiaonan Zhou, Yifei Liu, Jue Hu, Jing Zhang, Min Ren, Gang Ji, Xu Cai, Rui Bi

**Affiliations:** Department of Pathology, Fudan University Shanghai Cancer Center, Department of Oncology, Shanghai Medical College, Fudan University, 270 Dong’an Road, Shanghai 200032, China; zhouxn0909@163.com (X.Z.); 22211230050@m.fudan.edu.cn (Y.L.); hjjean1995@163.com (J.H.); iawguu@126.com (J.Z.); 18616707840@163.com (M.R.); jigang_2016@163.com (G.J.); caixu66@163.com (X.C.)

**Keywords:** ovarian clear cell carcinoma, clinicopathological features, TERT promoter mutation, prognosis

## Abstract

**Background:** Ovarian clear cell carcinoma (OCCC) is characterized by chemoresistance and poor prognosis in advanced or recurrent cases. This study aimed to find specific prognostic markers for OCCC. **Methods:** We analyzed 169 OCCC patients for clinicopathological features. TERT promoter and PIK3CA mutations were assessed by Sanger sequencing, and immunohistochemistry for ARID1A, HDAC6, Cyclin E1, and p53 was performed on tissue microarrays. Survival analysis was conducted using Kaplan–Meier and Cox regression models. **Results:** The -124C>T TERT promoter mutation was associated with longer OS and PFS and was an independent predictor of favorable OS. This mutation correlated with lower CA125 levels and higher SNP frequency. p53 mutations indicated advanced disease, bilateral tumors, reduced Cyclin E1, and poor prognosis. Low HDAC6 expression was linked to worse PFS. Mutual exclusivity was observed between PIK3CA exon 20 mutations and SNPs. **Conclusions:** The -124C>T TERT promoter mutation may serve as a favorable prognostic marker in OCCC, while p53 mutations and reduced HDAC6 expression are associated with poor outcomes.

## Simple Summary

Ovarian clear cell carcinoma is a rare but aggressive type of ovarian cancer that often responds poorly to standard chemotherapy, leading to unfavorable outcomes. This study aimed to identify biological markers that could help predict disease progression and guide treatment decisions. We analyzed tumor samples from 169 patients to assess specific gene mutations and protein expression levels. We found that patients with a TERT promoter mutation tended to have longer survival, slower disease progression, and lower levels of a common blood marker used to monitor cancer. In contrast, patients with mutations in the p53 gene or reduced levels of a protein called HDAC6 experienced worse outcomes. These findings suggest that testing for these genetic and protein changes could help doctors better predict disease prognosis and tailor treatment strategies. This research may contribute to improving survival and quality of life for patients with ovarian clear cell carcinoma.

## 1. Introduction

Ovarian cancer (OC), one of the most aggressive and lethal malignancies of the female reproductive system, is primarily characterized by a lack of specific early symptoms, often leading to diagnosis at an advanced stage [1]. It ranks as the sixth leading cause of cancer-related mortality among women, with over 210,000 new cases reported annually [2,3]. Despite advances in surgery, chemotherapy, and targeted therapies, the overall survival rate for OC remains poor [4]. Recent developments in targeted therapies for OC have shown promise, with several approaches currently under investigation or in clinical trials. Patients harboring BRAF mutations or dysregulation of the MAPK and PI3K pathways, particularly those with low-grade serous ovarian carcinoma, may significantly benefit from BRAF or MEK inhibitors [5]. In advanced high-grade OC, the identification of homologous recombination deficiency (HRD) status could expand the population eligible for poly (ADP-ribose) polymerase (PARP) inhibitors [6]. Therefore, molecular profiling of tumor-specific biomarkers is essential for guiding accurate diagnosis and optimizing individualized treatment strategies.

Ovarian clear cell carcinoma (OCCC) is the second most common epithelial ovarian cancer and has increased significantly in recent years in Southeast Asian populations, with a substantial incidence of 30% in Japan [7,8]. OCCC is characterized by distinct clinicopathological features and molecular alterations, frequently harboring somatic mutations in ARID1A, PIK3CA, and the TERT promoter (TERTp), often in association with endometriosis [9,10]. Consistent with the standard treatment for high-grade serous ovarian cancer, staging surgery or optimal cytoreduction combined with chemotherapy is the major therapeutic approach for treating OCCC [11]. Despite favorable clinical outcomes in early-stage patients, patients with advanced or recurrent OCCC were resistant to chemotherapy and had poor prognoses [12].

Despite advances in molecular profiling, OCCC lacks reliable prognostic markers, and the clinical significance of high-frequency mutations remains controversial [13,14]. Previous investigations have suggested adverse effects in patient outcomes to be associated with abnormal expression of histone deacetylase 6 (HDAC6), Cyclin E1, and p53 [15,16,17]. However, these findings require further investigation. In this study, we conducted a comprehensive analysis of OCCC to evaluate the relationship between clinicopathological features and their prognostic significance.

## 2. Materials and Methods

### 2.1. Patients and Tissue Samples

This retrospective study included 176 patients diagnosed with OCCC who were treated at Fudan University Shanghai Cancer Center (FUSCC) between January 2000 and December 2015. After excluding cases lacking complete clinicopathological data or follow-up information, a total of 169 patients were included in the final analysis. Clinical information was collected from electronic medical records, including age at diagnosis, tumor size, laterality, presence of ascites, preoperative serum CA125 levels, and disease stage according to the 2014 International Federation of Gynecology and Obstetrics (FIGO) guidelines [18]. The presence of endometriosis (ovarian or extra-ovarian) was also assessed. Patients were followed up until May 2023 through outpatient records or telephone interviews. Overall survival (OS) was defined as the time from initial surgery to death from any cause, and progression-free survival (PFS) was defined as the time from initial surgery to documented recurrence or progression.

### 2.2. Tissue Microarray (TMA) Construction

Formalin-fixed, paraffin-embedded (FFPE) tumor tissues from 169 OCCC cases were used to construct tissue microarrays (TMAs) with three representative 1 mm cores extracted from each specimen, ensuring standardized immunohistochemical (IHC) analysis, as previously reported [19].

### 2.3. Immunohistochemistry

Immunohistochemical staining was performed on TMA sections using standard protocols [19]. We performed immunohistochemical staining for ARID1A, p53, HDAC6, and Cyclin E1. The details of the antibodies used are summarized in Supplementary Table S2. Staining was visualized using the DAB chromogen and counterstained with hematoxylin. Immunohistochemical scoring was independently performed by two experienced pathologists, with discrepancies resolved by consensus. In summary, ARID1A deficiency was defined as the complete absence of nuclear staining, with no detectable staining in tumor cells, and the nuclei of stromal cells with brown staining as an internal positive control [20]. HDAC6 expression was scored using a histologic score (H score), as previously described [21], with 0 (0%), weak (1+, 1–50%), moderate (2+, 51–80%), or marked (3+, 81–100%) based on the percentage and intensity of brown staining localized within the cytoplasm [16]. Abnormal Cyclin E1 expression was characterized by diffuse and strong nuclear immunoreactivity observed in >80% of tumor cells [22]. Wild-type p53 was defined as showing weak or absent nuclear staining, indicating normal protein levels. In contrast, abnormal p53 mutant expression was defined as overexpression (strong nuclear expression involving >80% of tumor cell nuclei), or complete absence of expression in tumor cell nuclei with retained internal control, or unequivocal cytoplasmic expression (absence of staining due to truncating mutations or deletions) [23].

### 2.4. DNA Extraction and Mutation Analysis

We also extracted DNA from 3-µm sections with a QIAGEN DNA FFPE Tissue Kit (Qiagen, Shanghai, China), according to the manufacturer’s protocol. The annealing temperature for all pairs of primers was 56 °C. Sanger DNA sequencing was performed by Shenggong Bioengineering Co., Ltd. (Shanghai, China), and the sequencing data was analyzed using SnapGene version 6.0.2 software. All the mutations detected were confirmed by resequencing. The primers used for PIK3CA exon 9 and exon 20, as well as for the TERT promoter, are listed in Supplementary Table S3. All primers were synthesized by Tsingke Biotechnology Co., Ltd (Shanghai, China). Due to the relatively long storage of tissues, TERT promoter mutations were successfully analyzed in 87 cases, the rs2853669 single nucleotide polymorphism (SNP) in 81 cases, and mutations in exons 9 and 20 of PIK3CA in 107 and 109 cases, respectively.

### 2.5. Statistical Analysis

Age and tumor diameter were summarized as medians with interquartile ranges (IQRs), and other variables were described as counts and percentages using the R package TableOne. Group comparisons were performed using the Chi-square test or Fisher’s exact test for categorical variables, and the Mann–Whitney U test or Kruskal–Wallis test for continuous variables. To identify independent prognostic factors, all variables with *p* < 0.05 in univariate Cox regression analysis were included in multivariate Cox regression models. Hazard ratios (HRs) with 95% confidence intervals (CIs) were calculated. PFS and OS were estimated using the Kaplan–Meier method and compared using the log-rank test. A two-sided *p* < 0.05 was considered statistically significant. All analyses were performed using SPSS v25 and R v4.2.2.

## 3. Results

### 3.1. Patient Characteristics and Clinicopathological Factors

**Figure 1:**
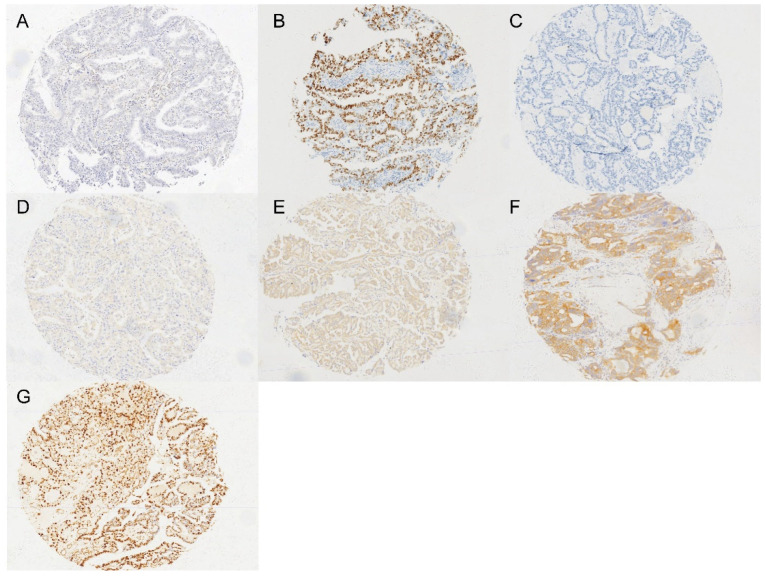
Immunohistochemical staining of ARID1A (**A**), p53 abnormal expression with diffuse strong staining (**B**), p53 abnormal expression with complete absence of staining (**C**), HDAC6 with weak staining score as 1+ (**D**), HDAC6 with moderate staining score as 2+ (**E**), HDAC6 with strong staining score as 3+ (**F**), and overexpression of Cyclin E1 (**G**) in OCCC. The images were captured at 40× magnification.

In our cohort, the median age was 53 years (range, 26–83 years), and the median tumor size was 11 cm (range, 2–35 cm). The majority of tumors were FIGO stage I/II (67%, 113/169) and unilateral (79%, 134/169). A total of 37% (62/169) of patients had endometriosis, and half of the patients (50%, 84/169) had ascites. Preoperative serum CA125 levels were elevated in nearly 80% (116/169) of patients. ARID1A deficiency was detected in 54% (70/129) of the patients (Figure 1A). Abnormal p53 expression was observed in 15% (25/165) of patients (Figure 1B,C). HDAC6 expression was scored as 1+, 2+, and 3+ in 22% (37/166), 24% (40/166), and 18% (30/166) of cases, respectively (Figure 1D–F). Cyclin E1 was overexpressed in 23% (38/167) of OCCC patients (Figure 1G).

The percentage of patients with PIK3CA mutations was 56% (61/109), in which the exon 9 and exon 20 mutation rates were 48% (51/107) and 18% (20/109), respectively. Mutations at the -124, -138, and -146 sites in the TERT promoter were identified in 22% (19/87), 1% (1/87), and 5% (4/87) of cases, respectively. The rs2853669 SNP at -245 bp in the TERT promoter was detected in 81 patients, with 62% (50/81) carrying the variant allele. The clinicopathological characteristics are summarized in Supplementary Table S1.

### 3.2. Prognostic Factors in OCCC

Patients aged ≥60 years had a shorter PFS than those aged <60 years at the time of diagnosis (Figure S2A). Patients with early stage, unilateral tumors, no ascites, a normal preoperative CA125 level, and wild-type p53 had better OS and PFS than patients with advanced-stage disease, bilateral tumors, ascites, a higher preoperative CA125 level, and abnormal expression of p53 (Figures S1C–G and S2C–G). In our cohort, no significant correlations were found between tumor size, ARID1A, Cyclin E1, HDAC6, history of endometriosis, PIK3CA exon 9 and exon 20 mutations, the -146C>T mutation, or SNP status of TERTp with OS and PFS. Despite the lack of statistically significant findings, patients with the -146C>T mutation tended to have worse OS and PFS (Figures S1B,H–P and S2B,J–R).

**Figure 2:**
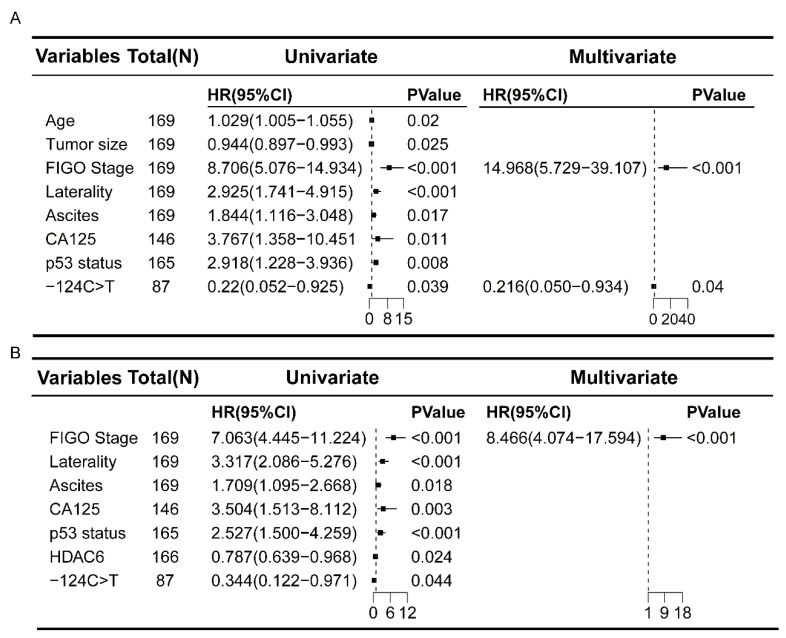
Univariate analysis and multivariate Cox regression analysis of clinicopathological factors associated with OCCC (attached with forest plots). (**A**) Univariate and multivariate Cox regression analyses for OS. (**B**) Univariate and multivariate Cox regression analyses for PFS.

**Figure 3:**
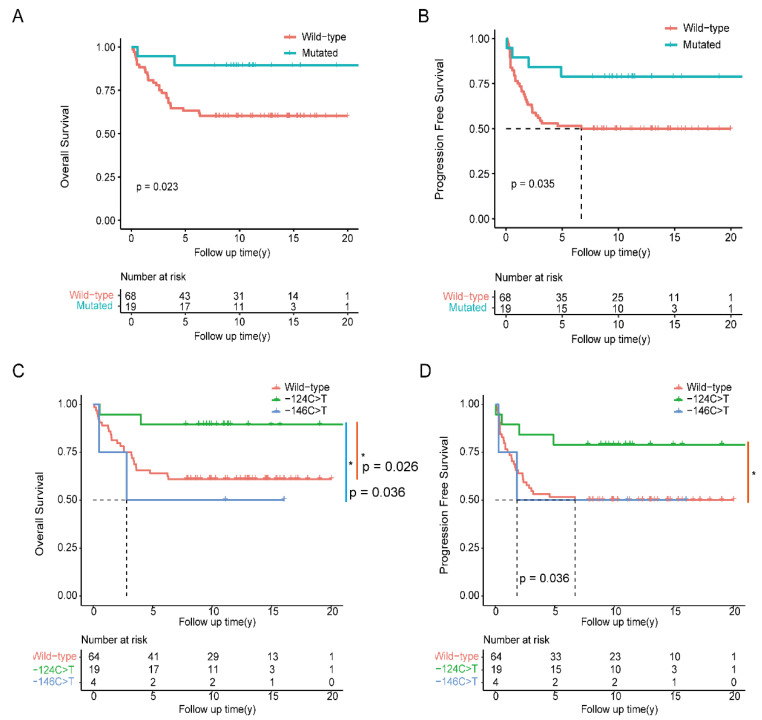
Kaplan–Meier survival curves for OS and PFS in OCCC patients. (**A**) Kaplan–Meier analysis of OS in patients with the -124C>T mutation. (**B**) Kaplan–Meier analysis of PFS in patients with the -124C>T mutation. (**C**) Kaplan–Meier analysis of OS in patients with the wild-type, -124C>T and -146C>T mutations. (**D**) Kaplan–Meier analysis of OS in patients with the wild-type, -124C>T and -146C>T mutations. *: *p* < 0.05. The significance of the prognostic value was tested by the log-rank test.

All the above variables that were significant in univariate Cox regression analysis (*p* < 0.05) were subjected to multivariate Cox regression analysis. The stage and -124 C>T mutation were found to be independent prognostic factors for OS in OCCC patients. Multivariate analyses confirmed the negative effect of stage and the positive effect of the -124 C>T mutation on patient prognosis, with HR values for death of 14.968 (95% CI: 5.729–39.107; *p* < 0.001) and 0.216 (95% CI: 0.050–0.934; *p* = 0.040), respectively (Figure 2A). In addition, with an HR for disease progression of 8.466 (95% CI: 4.074–17.594; *p* < 0.001), stage was an independent prognostic factor for PFS in OCCC patients (Figure 2B). Kaplan–Meier survival analysis revealed that patients with the -124C>T mutation had significantly better OS (*p* = 0.023) and PFS (*p* = 0.035) than those with the -124C>T wild type (Figure 3A,B). After adjusting for stage in the multivariate analysis, patients with the -24C>T mutation had longer OS only in advanced stages (III and IV), while no significant OS difference was observed between mutated and wild-type TERT promoter groups in early stages (I and II) (Figure S1H,I).

To determine the potential impact of different TERT promoter mutations on patient prognosis, we investigated the association between TERT promoter mutation status and OS and PFS. The OS and PFS of OCCCs with the -124 C>T mutation in TERTp were significantly longer than those with the wild-type gene (*p* = 0.026 and *p* = 0.036, respectively) (Figure 3C,D). Compared with patients carrying the -146 C>T mutation, patients with the -124 C>T mutation had substantially longer OS (*p* = 0.036) (Figure 3C). Although the difference was not significant, patients carrying the -146 C>T mutation tended to have worse OS (Figure 3C).

### 3.3. Correlations Between TERTp Mutation and Other Clinicopathological Factors in OCCC

Patients with the -124C>T mutation were more likely to have a normal preoperative serum CA125 level (*p* = 0.017), a higher incidence of SNPs (*p* = 0.014), and a poorer prognosis (*p* = 0.035 for progression and *p* = 0.026 for survival) than wild-type patients (Table 1). We also investigated other mutations in the TERTp, revealing a significant mutual exclusivity between SNP status and PIK3CA exon 20 mutations (*p* = 0.030) (Table 1). Further investigation of the associations between clinicopathological factors and the expression or mutation status of ARID1A, p53, HDAC6, Cyclin E1, and PIK3CA exons 9 and 20 revealed several critical findings. Mutations in p53 were significantly associated with bilaterality in OCCC (*p* = 0.009), elevated preoperative CA125 levels (*p* = 0.011), advanced-stage disease (*p* = 0.034), and reduced Cyclin E1 expression (*p* = 0.032). Importantly, p53 mutations were associated with worse disease progression (*p* = 0.002) and survival (*p* = 0.015). Additionally, lower HDAC6 expression correlated with worse disease progression (*p* = 0.047). The correlations are summarized in Table 1.

## 4. Discussion

In this study, we assessed the prognostic significance of clinicopathological factors in OCCC patients treated at our institution over a period of 23 years. We observed that the rates of ARID1A loss expression and somatic driver mutations in PIK3CA and TERTp were 54% (70/129), 56% (61/109) and 26% (23/87), respectively. Large-scale sequencing of putative somatic driver mutations in 401 OCCC tumors revealed mutation rates of 49% in PIK3CA and 20% in TERTp, which were slightly lower than those observed in our study [10]. In general, TERTp mutations correlate with increased TERT mRNA levels and increased telomerase activity [24]. The primary TERTp mutations occur at positions 1,295,228 (-124 C>T) and 1,295,250 (-146 C>T) [25]. Consistent with the findings of previous studies, the -124 C>T mutation predominated among the TERTp mutations, with 21.8% mutation frequency [26,27]. The -124 C>T mutation and stage were found to be independent prognostic factors for OS in our cohort. Patients with the -124 C>T mutation had significantly longer PFS and OS than wild-type patients and were more likely to have a normal preoperative serum CA125 level, a greater incidence of SNPs, and were less likely to relapse.

The clinical significance of TERTp mutations in OCCC remains controversial. While TERTp mutations, such as the -124C>T hotspot, are associated with elevated TERT mRNA expression and increased telomerase activity [28,29], their prognostic implications remain conflicting. Huang et al. identified TERTp mutations as independent predictors of shorter disease-free survival (DFS) and OS in early-stage OCCC patients [30]. Yoo et al. similarly demonstrated their association with recurrence, platinum resistance, and DFS [31]. In contrast, Kobayashi et al. observed prolonged PFS in the ‘enhancer-type’ group, which includes TERTp hotspot mutations, compared to wild-type or mutation subgroups [32]. Furthermore, no significant differences in OS were noted in studies by Nishikimi et al. and Wu et al. [28,33].

In our study, patients with the -124C>T mutation exhibited significantly longer PFS and OS than their wild-type counterparts. This favorable prognosis may stem from multiple factors. Functional studies suggest that the impact of TERTp mutations on survival is context-dependent, potentially influenced by SNPs like rs2853669. This variant allele can attenuate TERT promoter activity in the presence of -124C>T, mitigating its oncogenic effects and improving prognosis [34]. Consistent with this, most -124C>T mutations in our cohort frequently co-occurred with SNPs. TERTp mutations may reshape tumor immunogenicity. Transcriptomic analyses have linked TERTp mutations to epithelial-to-mesenchymal transition (EMT) and MAPK signaling signatures [35], pathways that are associated with immune activation. For instance, EMT-high tumors often demonstrate increased immune checkpoint gene expression, while gliomas with TERTp mutations show elevated PD-L1 levels [36]. These findings suggest that TERTp mutations may enhance tumor immunogenicity and augment anti-tumor immune responses. Furthermore, epigenetic factors may modulate the prognostic impact of TERTp mutations. In glioblastoma, MGMT promoter methylation significantly improves survival outcomes in TERTp-mutant tumors [37]. Additionally, functional differences between the -124C>T and -146C>T hotspot mutations could explain variations in clinical behavior, with the former exhibiting distinct promoter activity profiles [38,39]. Collectively, these findings underscore the complexity of TERTp mutations in OCCC, emphasizing their interactions with genetic alterations, immune microenvironment, and broader tumor biology. While the favorable prognosis observed in -124C>T-mutant patients in our study contrasts with prior reports, longer follow-up and in-depth analyses of molecular co-factors may provide critical insights.

ARID1A encodes an accessory subunit of the SWI/SNF chromatin remodeling complex, which exhibits a high frequency of mutations in OCCC [40]. In a comprehensive study involving 1432 patients with endometrium-associated gynecological cancers, loss of ARID1A expression predicted shorter PFS [41]. In our cohort study, the absence of ARID1A expression did not show statistical significance in relation to PFS and OS (Figures S1K and S2I). Studies have demonstrated that ARID1A directly repressed HDAC6 gene transcription, and mutations in ARID1A and TP53 were mutually exclusive [42,43]. ARID1A deficiency tended to correlate with increased HDAC6 expression and a higher incidence of p53 mutations in our cohort, though without reaching statistical significance (Table 1). Additionally, high HDAC6 expression had an adverse effect on PFS in patients with ARID1A loss [16]. Interestingly, higher HDAC6 expression was linked to better PFS (Figure S2J). Emerging evidence indicates that chemotherapy can modulate HDAC6 expression [44]. In OCCC, the prognostic significance of HDAC6 expression remains to be validated in larger patient cohorts. Further studies are warranted to assess HDAC6 expression before and after chemotherapy and to clarify its association with clinical outcomes. Furthermore, PIK3CA mutations were significantly associated with favorable OS in a cohort of 56 Japanese patients with OCCC [45]. In our cohort, there were no prognostic differences between cases with and without PIK3CA exon 9 or exon 20 mutations among 109 patients (Figures S1N,O and S2L,M). Huang et al. reported a significant correlation between the loss of ARID1A expression and PI3K-Akt pathway activation [46]. Despite the lack of statistical significance, loss of ARID1A expression tends to coexist with PIK3CA exon 9 mutation but is mutually exclusive with exon 20 mutation (Table 1). Previous studies have revealed that TERTp mutations were mutually exclusive with the loss of ARID1A expression or PIK3CA mutations in OCCC [28,30]. Although no significant correlation between ARID1A and TERTp mutations was identified, our cohort revealed a mutually exclusive pattern between SNP status and PIK3CA exon 20 mutations (Table 1, *p* = 0.030).

Cyclin E1 is a highly conserved cell cycle regulatory protein, and its overexpression is closely correlated with TERTp mutations and poor outcomes in stage I patients [22]. In epithelial ovarian cancer, high Cyclin E1 expression is associated with poor prognosis, and strong nuclear Cyclin E1 serves as an independent predictor of worse PFS. Cyclin D1 shows low nuclear and cytoplasmic expression, and reduced cytoplasmic cyclin D1 correlates with shorter OS [17]. These correlations were not observed in our cohort; however, Cyclin E1 expression was mutually exclusive with mutant p53 expression (Figures S1M and S2K, Table 1, *p* = 0.032). The differing conclusions may stem from differences in patient selection or population exposure estimation and the different numbers of patients enrolled. To better understand the roles of ARID1A, PIK3CA, TERT, TP53, HDAC6, and Cyclin E1 in ovarian cancer, we summarized their mutation frequencies and expression patterns across different histological subtypes, as presented in Supplementary Table S4.

This study acknowledges certain limitations. Despite a large sample size and lengthy follow-up, some FFPE samples were of inadequate quality for effective mutation analysis. Moreover, the study did not consider the diversity of postoperative adjuvant treatments in OCCC, focusing solely on the relationships between clinical prognostic factors rather than treatment impacts. Future research will aim to close these gaps and deepen insights into OCCC prognosis.

## 5. Conclusions

In conclusion, we assessed the prognostic value of common gene mutations and clinicopathologic factors in OCCC and found that the -124 C>T mutation of TERTp represents a favorable prognostic factor for OS in OCCC patients. This finding may have great potential to advance precision medicine, enabling personalized treatments and other targeted interventions. However, further study is needed to elucidate the molecular mechanisms by which TERTp mutations influence the prognosis of patients with OCCC.

## Figures and Tables

**Table 1:** The correlations between TERTp -124C>T mutation and clinicopathological factors in OCCC.

	-124C>T Mutation			-146C>T Mutation			-138C>T Mutation			SNP Statue			p53			ARID1A Expression			HDAC6					CyclinE1			PIK3CA Exon9 Mutation			PIK3CA Exon20 Mutation		
	Wild-type	Mutant	*p*	Wild-type	Mutant	*p*	Wild-type	Mutant	*p*	Noncarrier	Carrier	*p*	Wild-type	Mutant	*p*	Intact	Deficient	*p*	0	1+	2+	3+	*p*	Normal	Abnormal	*p*	Wild-type	Mutant	*p*	Wild-type	Mutant	*p*
Age			0.061			0.454			1			0.76			0.419			0.647					0.335			0.11			0.824			0.149
<60 y	56 (75)	19 (25)		72 (96)	3 (4)		74 (99)	1 (1)		27 (39)	42 (61)		113 (86)	18 (14)		50 (47)	57 (53)		44 (33)	32 (24)	34 (26)	22 (17)		99 (74)	34 (26)		41 (51)	39 (49)		64 (78)	18 (22)	
≥60 y	12 (100)	0 (0)		11 (92)	1 (8)		12 (100)	0 (0)		4 (33)	8 (67)		27 (79)	7 (21)		9 (41)	13 (59)		15 (44)	5 (15)	6 (18)	8 (23)		30 (88)	4 (12)		15 (56)	12 (44)		25 (93)	2 (7)	
Tumor size			1			1			1			0.128			0.322			0.332					0.983			0.4			0.505			0.395
<15 cm	51 (79)	14 (21)		62 (95)	3 (5)		64 (99)	1 (1)		26 (43)	34 (57)		102 (83)	21 (17)		40 (43)	53 (57)		44 (36)	27 (22)	29 (24)	23 (18)		98 (79)	26 (21)		40 (50)	40 (50)		68 (84)	13 (16)	
≥15 cm	17 (77)	5 (23)		21 (96)	1 (4)		22 (100)	0 (0)		5 (24)	16 (76)		38 (90)	4 (10)		19 (53)	17 (47)		15 (35)	10 (23)	11 (26)	7 (16)		31 (72)	12 (28)		16 (59)	11 (41)		21 (75)	7 (25)	
Laterality			0.505			0.153			1			0.39			**0.009**			0.179					0.65			0.825			0.801			0.517
Unilaterality	54 (76)	17 (24)		69 (97)	2 (3)		70 (99)	1 (1)		23 (35)	42 (65)		119 (89)	15 (11)		51 (49)	53 (51)		44 (33)	30 (23)	34 (26)	24 (18)		103 (77)	31 (23)		46 (52)	43 (48)		73 (80)	18 (20)	
Bilaterality	14 (88)	2 (12)		14 (88)	2 (12)		16 (100)	0 (0)		8 (50)	8 (50)		21 (68)	10 (32)		8 (32)	17 (68)		15 (44)	7 (20)	6 (18)	6 (18)		26 (79)	7 (21)		10 (56)	8 (44)		16 (89)	2 (11)	
CA125			**0.017**			1			0.211			0.344			**0.011**			0.828					0.163			0.804			0.216			1
Normal	8 (50)	8 (50)		15 (94)	1 (6)		15 (94)	1 (6)		3 (23)	10 (77)		30 (100)	0 (0)		12 (46)	14 (54)		10 (34)	4 (13)	7 (23)	9 (30)		23 (77)	7 (23)		8 (40)	12 (60)		16 (80)	4 (20)	
Abnormal	50 (83)	10 (17)		57 (95)	3 (5)		60 (100)	0 (0)		24 (41)	34 (59)		90 (79)	24 (21)		44 (49)	46 (51)		43 (37)	28 (25)	29 (25)	15 (13)		91 (79)	24 (21)		40 (56)	31 (44)		58 (80)	15 (20)	
Ascites			1			0.349			1			0.497			0.83			1					0.861			0.36			0.848			1
Absent	35 (78)	10 (22)		44 (98)	1 (2)		44 (98)	1 (2)		14 (34)	27 (66)		72 (86)	12 (14)		31 (46)	37 (54)		29 (35)	20 (24)	18 (22)	16 (19)		63 (74)	22 (26)		31 (53)	27 (47)		48 (81)	11 (19)	
Present	33 (79)	9 (21)		39 (93)	3 (7)		42 (100)	0 (0)		17 (43)	23 (57)		68 (84)	13 (16)		28 (46)	33 (54)		30 (36)	17 (21)	22 (26)	14 (17)		66 (80)	16 (20)		25 (51)	24 (49)		41 (82)	9 (18)	
Endometriosis			0.274			0.294			0.345			1			1			0.59					0.38			0.567			0.107			1
Absent	47 (83)	10 (17)		53 (93)	4 (7)		57 (100)	0 (0)		20 (38)	33 (62)		87 (85)	16 (15)		34 (44)	44 (56)		41 (39)	25 (24)	23 (22)	16 (15)		83 (79)	22 (21)		40 (59)	28 (41)		56 (81)	13 (19)	
Present	21 (70)	9 (30)		30 (100)	0 (0)		29 (97)	1 (3)		11 (39)	17 (61)		53 (86)	9 (14)		25 (49)	26 (51)		18 (30)	12 (20)	17 (28)	14 (22)		46 (74)	16 (26)		16 (41)	23 (59)		33 (83)	7 (17)	
Stage			0.408			0.58			1			0.621			**0.034**			0.337					0.338			0.566			0.668			1
I–II	46 (75)	15 (25)		59 (97)	2 (3)		60 (98)	1 (2)		20 (36)	36 (64)		100 (89)	12 (11)		44 (49)	46 (51)		36 (32)	26 (23)	27 (24)	24 (21)		85 (76)	27 (24)		39 (51)	38 (49)		65 (82)	14 (18)	
III–IV	22 (85)	4 (15)		24 (92)	2 (8)		26 (100)	0 (0)		11 (44)	14 (56)		40 (76)	13 (24)		15 (38)	24 (62)		23 (43)	11 (21)	13 (25)	6 (11)		44 (80)	11 (20)		17 (57)	13 (43)		24 (80)	6 (20)	
Disease Progression			**0.035**			1			1			0.823			**0.002**			0.6					**0.047**			0.854			1			0.618
No	34 (69)	15 (31)		47 (96)	2 (4)		48 (98)	1 (2)		17 (40)	26 (60)		83 (93)	6 (7)		34 (48)	37 (52)		25 (28)	21 (24)	20 (23)	22 (25)		68 (76)	21 (24)		32 (53)	29 (47)		52 (84)	10 (16)	
Yes	34 (90)	4 (10)		36 (95)	2 (5)		38 (100)	0 (0)		14 (37)	24 (63)		57 (75)	19 (25)		25 (43)	33 (57)		34 (44)	16 (20)	20 (26)	8 (10)		61 (78)	17 (22)		24 (52)	22 (48)		37 (79)	10 (21)	
Survival Statue			**0.026**			0.598			1			0.641			**0.015**			0.353					0.061			0.705			1			
Alive	41 (71)	17 (29)		56 (97)	2 (3)		57 (98)	1 (2)		21 (40)	31 (60)		93 (90)	10 (10)		42 (49)	44 (51)		29 (28)	27 (26)	27 (26)	21 (20)		79 (76)	25 (24)		37 (53)	33 (47)		75 (81)	18 (19)	
Dead	27 (93)	2 (7)		27 (93)	2 (7)		29 (100)	0 (0)		10 (35)	19 (65)		47 (76)	15 (24)		17 (40)	26 (60)		30 (48)	10 (16)	13 (21)	9 (15)		50 (79)	13 (21)		19 (51)	18 (49)		12 (86)	2 (14)	
-124C>T mutation						0.572			0.218																				0.262			
Wild-type				64 (94)	4 (6)		68 (100)	0 (0)																								
Mutant				19 (100)	0 (0)		18 (95)	1 (5)																								
-146C>T mutation			0.572																													
Wild-type	64 (77)	19 (23)																														
Mutant	4 (100)	0 (0)																														
-138C>T mutation			0.218			1																										
Wild-type	68 (79)	18 (21)		82 (95)	4 (5)																											
Mutant	0 (0)	1 (100)		1 (100)	0 (0)																											
SNP			**0.014**			1			1																							
Noncarrier	30 (97)	1 (3)		30 (97)	1 (3)		31 (100)	0 (0)																								
Carrier	38 (76)	12 (24)		47 (94)	3 (6)		49 (98)	1 (2)																								
p53			0.725			1			1			1																				
Wild-type	56 (77)	17 (23)		69 (95)	4 (5)		72 (99)	1 (1)		26 (39)	41 (61)																					
Mutant	12 (86)	2 (14)		14 (100)	0 (0)		14 (100)	0 (0)		5 (36)	9 (64)																					
ARID1A			0.266			0.267						0.175			0.192																	
Intact	22 (82)	5 (18)		27 (100)	0 (0)					12 (48)	13 (52)		53 (91)	5 (9)																		
Deficient	27 (68)	13 (32)		37 (93)	3 (7)					10 (28)	26 (72)		56 (82)	12 (18)																		
HDAC6			0.461			0.641			1			0.353			0.181			0.113														
0	20 (71)	8 (29)		26 (93)	2 (7)		27 (96)	1 (4)		11 (42)	15 (58)		48 (83)	10 (17)		21 (57)	16 (43)															
1+	18 (78)	5 (22)		23 (100)	0 (0)		23 (100)	0 (0)		10 (48)	11 (52)		29 (83)	6 (17)		13 (38)	21 (62)															
2+	18 (90)	2 (10)		19 (95)	1 (5)		20 (100)	0 (0)		4 (21)	15 (79)		31 (80)	8 (20)		18 (53)	16 (47)															
3+	11 (73)	4 (27)		14 (93)	1 (7)		15 (100)	0 (0)		5 (36)	9 (64)		29 (97)	1 (3)		7 (29)	17 (71)															
Cyclin E1			0.754			0.582			1			0.559			**0.032**			0.102					0.134									
Normal	54 (77)	16 (23)		66 (94)	4 (6)		69 (99)	1 (1)		24 (36)	42 (64)		105 (81)	24 (19)		40 (42)	56 (58)		49 (39)	30 (24)	27 (21)	21 (16)										
Abnormal	14 (82)	3 (18)		17 (100)	0 (0)		17 (100)	0 (0)		7 (47)	8 (53)		35 (97)	1 (3)		19 (59)	13 (41)		9 (24)	6 (16)	13 (35)	9 (25)										
PIK3CA Exon9			0.213			0.544			1			1			0.568			0.262					0.421			0.175						
Wild-type	29 (76)	9 (24)		35 (92)	3 (8)		37 (97)	1 (3)		11 (31)	24 (69)		49 (89)	6 (11)		20 (49)	21 (51)		22 (39)	10 (18)	13 (23)	11 (20)		45 (82)	10 (18)							
Mutant	10 (59)	7 (41)		17 (100)	0 (0)		17 (100)	0 (0)		5 (36)	9 (64)		42 (84)	8 (16)		14 (34)	27 (66)		13 (26)	14 (28)	14 (28)	9 (18)		35 (69)	16 (31)							
PIK3CA Exon20			0.475			0.113			1			**0.03**			1			0.148					0.538			0.083			0.45			
Wild-type	30 (68)	14 (32)		43 (98)	1 (2)		43 (98)	1 (2)		10 (26)	29 (74)		75 (86)	12 (14)		25 (37)	42 (63)		29 (33)	22 (24)	21 (24)	17 (19)		70 (79)	18 (21)		45 (51)	44 (49)				
Mutant	10 (83)	2 (17)		10 (83)	2 (17)		12 (100)	0 (0)		7 (64)	4 (36)		18 (90)	2 (10)		9 (60)	6 (40)		8 (42)	2 (10)	6 (32)	3 (16)		12 (60)	8 (40)		11 (61)	7 (39)				

## Data Availability

The data presented in this study are available on request from the corresponding authors.

## Supplementary Materials

The following supporting information can be downloaded at https://www.mdpi.com/article/10.3390/curroncol32080422/s1, Figure S1. Kaplan–Meier survival curves for OS of ovarian clear cell carcinoma (OCCC) patients. A. Kaplan–Meier analysis of age. B. Kaplan–Meier analysis of tumor size. C. Kaplan–Meier analysis of FIGO stage. D. Kaplan–Meier analysis of tumor laterality. E. Kaplan -Meier analysis of the history of ascites. F. Kaplan–Meier analysis of the preoperative serum CA125 level. G. Kaplan–Meier analysis of the p53 expression. H. Kaplan–Meier analysis of the -124C>T mutation for early-stage patients. I. Kaplan–Meier analysis of the -124C>T mutation for advanced-stage patients. J. Kaplan–Meier analysis of the history of endometriosis. K. Kaplan–Meier analysis of ARID1A expression. L. Kaplan–Meier analysis of HDAC6 expression. M. Kaplan–Meier analysis of CyclinE1 expression. N. Kaplan–Meier analysis of the mutation in PIK3CA exon 9. O. Kaplan–Meier analysis of the mutation in PIK3CA exon 20. P. Kaplan–Meier analysis of the -146C>T mutation of TERTp. Q. Kaplan–Meier analysis of the -138C>T mutation of TERTp. R. Kaplan–Meier analysis of the SNP statue of TERTp. Figure S2. Kaplan–Meier survival curves for PFS of ovarian clear cell carcinoma (OCCC) patients. A. Kaplan–Meier analysis of age. B. Kaplan -Meier analysis of tumor size. C. Kaplan–Meier analysis of FIGO stage. D. Kaplan -Meier analysis of tumor laterality. E. Kaplan–Meier analysis of the history of ascites. F. Kaplan–Meier analysis of the level of the preoperative serum CA125 level. G. Kaplan -Meier analysis of the p53 expression. H. Kaplan–Meier analysis of the history of endometriosis. I. Kaplan -Meier analysis of ARID1A expression. J. Kaplan -Meier analysis of HDAC6 expression. K. Kaplan–Meier analysis of CyclinE1 expression. L. Kaplan–Meier analysis of the mutation in PIK3CA exon 9. M. Kaplan–Meier analysis of the mutation in PIK3CA exon 20. N. Kaplan–Meier analysis of the -146C>T mutation of TERTp. O. Kaplan–Meier analysis of the -138C>T mutation of TERTp. P. Kaplan–Meier analysis of the SNP statue of TERTp. Table S1. The clinicopathological characteristics of 169 OCCCs. Table S2. List of antibodies were used in this study. Table S3. List of primers were used in this study. Table S4. Summary of Mutation Frequencies and Expression Patterns of ARID1A, PIK3CA, TERT, TP53, HDAC6, and Cyclin E1 Across Different Histological Subtypes of Ovarian Cancer.
