# Supplementary material for: The -124C>T Mutation of the TERT Promoter Indicates Favorable Prognosis in Ovarian Clear Cell Carcinoma: A Single Institutional Study in China
Source: Curr Oncol. 2025 Jul 27;32(8):422. doi: 10.3390/curroncol32080422 (PMC12384792; doi:10.3390/curroncol32080422)
Supplement: Supplementary file 1 [file curroncol-32-00422-s001.zip › Figure S2.pdf]

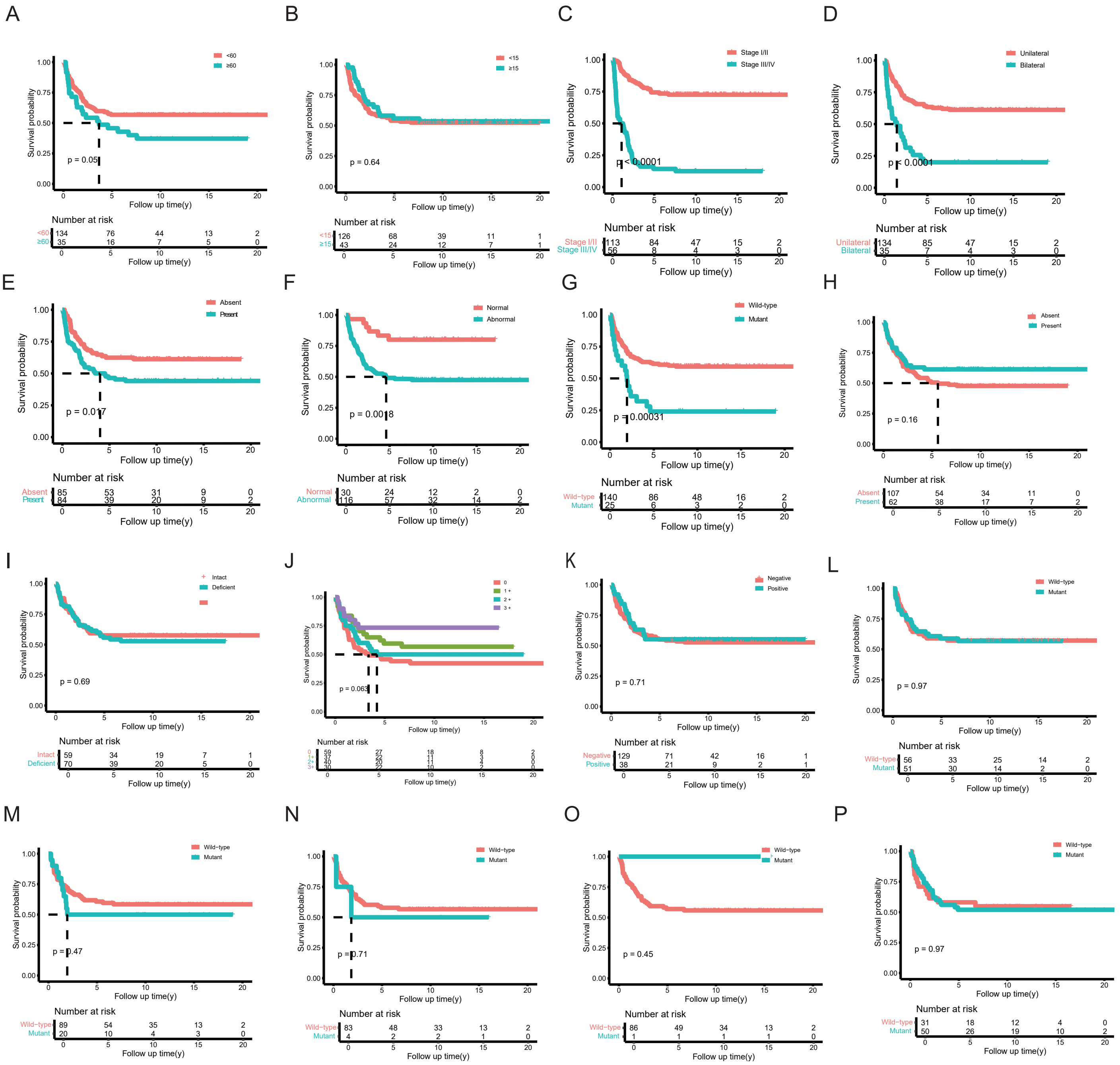

Figure S2. Kaplan–Meier survival curves for PFS of ovarian clear cell carcinoma (OCCC) patients. A. Kaplan–Meier analysis of age. B. Kaplan -Meier analysis of tumor size. C. Kaplan–Meier analysis of FIGO stage. D. Kaplan -Meier analysis of tumor laterality. E. Kaplan–Meier analysis of the history of ascites. F. Kaplan–Meier analysis of the level of the preoperative serum CA125 level. G. Kaplan -Meier analysis of the p53 expression. H. Kaplan–Meier analysis of the history of endometriosis. I. Kaplan -Meier analysis of ARID1A expression. J. Kaplan -Meier analysis of HDAC6 expression. K. Kaplan–Meier analysis of CyclinE1 expression. L. Kaplan–Meier analysis of the mutation in PIK3CA exon 9. M. Kaplan–Meier analysis of the mutation in PIK3CA exon 20. N. Kaplan–Meier analysis of the -146C>T mutation of TERTp. O. Kaplan–Meier analysis of the -138C>T mutation of TERTp. P. Kaplan–Meier analysis of the SNP statue of TERTp.
